# *Ralstonia solanacearum* Extracellular Polysaccharide Is a Specific Elicitor of Defense Responses in Wilt-Resistant Tomato Plants

**DOI:** 10.1371/journal.pone.0015853

**Published:** 2011-01-06

**Authors:** Annett Milling, Lavanya Babujee, Caitilyn Allen

**Affiliations:** Department of Plant Pathology, University of Wisconsin-Madison, Madison, Wisconsin, United States of America; University of Wisconsin-Milwaukee, United States of America

## Abstract

*Ralstonia solanacearum*, which causes bacterial wilt of diverse plants, produces copious extracellular polysaccharide (EPS), a major virulence factor. The function of EPS in wilt disease is uncertain. Leading hypotheses are that EPS physically obstructs plant water transport, or that EPS cloaks the bacterium from host plant recognition and subsequent defense. Tomato plants infected with *R. solanacearum* race 3 biovar 2 strain UW551 and tropical strain GMI1000 upregulated genes in both the ethylene (ET) and salicylic acid (SA) defense signal transduction pathways. The horizontally wilt-resistant tomato line Hawaii7996 activated expression of these defense genes faster and to a greater degree in response to *R. solanacearum* infection than did susceptible cultivar Bonny Best. However, EPS played different roles in resistant and susceptible host responses to *R. solanacearum*. In susceptible plants the wild-type and *ep*s^−^ mutant strains induced generally similar defense responses. But in resistant Hawaii7996 tomato plants, the wild-type pathogens induced significantly greater defense responses than the *ep*s^−^ mutants, suggesting that the resistant host recognizes *R. solanacearum* EPS. Consistent with this idea, purified EPS triggered significant SA pathway defense gene expression in resistant, but not in susceptible, tomato plants. In addition, the *ep*s^−^ mutant triggered noticeably less production of defense-associated reactive oxygen species in resistant tomato stems and leaves, despite attaining similar cell densities *in planta*. Collectively, these data suggest that bacterial wilt-resistant plants can specifically recognize EPS from *R. solanacearum*.

## Introduction

Plants resist many potential pathogens with low-amplitude innate immunity defenses that are triggered by recognition of microbe-associated molecular patterns (MAMPs) such as bacterial flagellin [1], [2]. R-gene mediated plant disease resistance typically involves much higher amplitude defense responses launched in response to pathogen effectors (avirulence factors) that the pathogen needs for full virulence and that the resistant plant has evolved to recognize [1]. The triggers and mechanisms of horizontal plant disease resistance are poorly understood, although this type of resistance is often stable and is widely deployed in agriculture [3].

As for many other plant diseases, resistance breeding is the best control for bacterial wilt (BW), a serious vascular disease caused by the soilborne bacterium *Ralstonia solanacearum* [4]. There is no single-gene resistance to BW in tomato, an economically important natural host of *R. solanacearum*. The most widely used resistance source is Hawaii7996 (H7996), a breeding line that carries at least five QTLs that together confer resistance to most pathogen strains via unknown mechanism(s) [5], [6], [7]. However, this horizontally-resistant line is not immune to the pathogen, and latent infections occur frequently (3). The defense signaling pathways triggered by BW disease development in tomato are not known, and these have direct implications for understanding and selecting BW-resistant germplasm. Thus, one aim of this study was to describe the kinetics of defense responses in susceptible and resistant tomato plants infected by two biologically distinct strains of *R. solanacearum*.

Extracellular polysaccharide (EPS) is a major virulence factor of *R. solanacearum* [8]. Site-directed mutants unable to synthesize EPS I, a heterogenous polymer of N-acetylated monosaccharides, are nearly avirulent and do not colonize plant xylem vessels as well as wild-type [9], [10]. *R. solanacearum* is a genetically diverse species complex, but the EPS structure is sufficiently well-conserved that an anti-EPS antibody can recognize all members of the group [11], [12]. EPS synthesis is regulated by the PhcA quorum sensing system such that it is produced abundantly at high cell densities in culture or when the bacterium grows in the confines of host plant xylem vessels [13]. However, it is not known how EPS contributes to BW disease development. It has been suggested that EPS directly causes wilting by physically blocking water flow in the densely-colonized xylem vessels of infected hosts [14]. It has also been hypothesized that the pathogen needs EPS to form biofilms on vessel surfaces during disease development; that EPS helps *R. solanacearum* survive desiccation or antibiosis in soil during periods away from host plants; and finally that EPS protects *R. solanacearum* from plant antimicrobial defenses by cloaking bacterial surface features that could be recognized by hosts [9], [14], [15], [16].

To test the latter hypothesis, we measured expression of defense genes and production of defensive reactive oxygen species (ROS) in susceptible and resistant tomato plants infected by wild-type and *eps^−^* mutants of two *R. solanacearum* strains. We found that *eps^−^* bacteria triggered similar defense signal pathway expression in a BW-susceptible tomato, undermining the cloaking hypothesis. Unexpectedly, BW-resistant H7996 plants expressed reduced defenses against the *eps^−^* strain, but they did activate the salicylic acid defense pathway in response to cell-free purified EPS. These results suggest that BW-resistant tomato plants recognize EPS, an abundantly-expressed and indispensible virulence factor of *R. solanacearum*.

## Results

### Temperate *R. solanacearum* strain UW551 breaks the BW resistance of H7996 tomato

**Figure 1:**
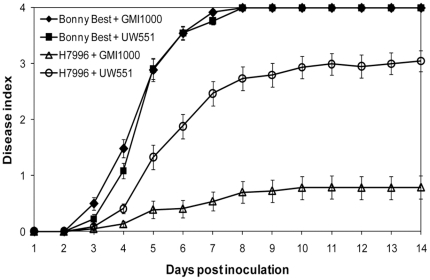
Virulence of *Ralstonia solanacearum* strains GMI1000 and UW551 on resistant and susceptible tomato plants. Unwounded susceptible (cv. Bonny Best) and horizontally resistant (H7996) tomato plants were soil-soak inoculated to a concentration of ∼1×10^8^ CFU/g soil and incubated at 28°C. Plants were rated daily on a 0 to 4 disease index scale where 0 = healthy and 4 = 100% wilted. Each point represents the mean disease index (± SE) for four independent experiments, each containing 16 plants per treatment.

*R. solanacearum* strains GMI1000 and UW551 were both highly virulent on susceptible tomato cv. Bonny Best (Figure 1). All inoculated plants were dead by 8 dpi and the strains had indistinguishable disease progress curves. In contrast, tomato breeding line H7996, a widely-used source of BW disease resistance, was quite resistant to tropical strain GMI1000; only 12% of the plants were dead by 14 dpi (Figure 1). However, H7996 was susceptible to *R. solanacearum* UW551, a typical sequevar 1 (Race 3 biovar 2) strain that causes losses in temperate zones and tropical highlands [17]. UW551 killed about 80% of H7996 plants within 14 dpi. The virulence of strains GMI1000 and UW551 was significantly different (*P*<0.001) on the resistant tomato plants.

### Tomato plants responded to *R. solanacearum* infection by upregulating marker genes for the salicylic acid (SA) and ethylene (ET) defense pathways

**Figure 2:**
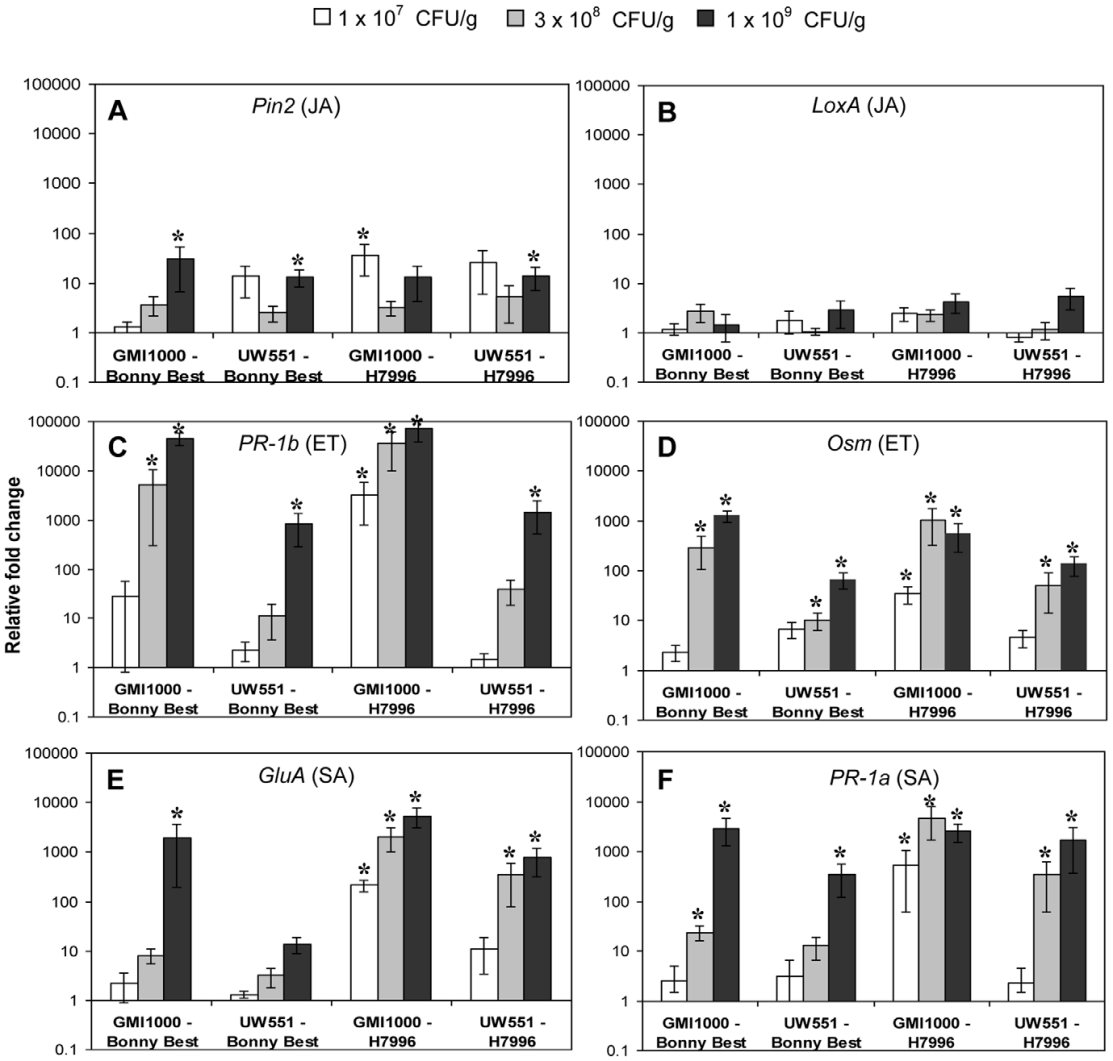
Expression of tomato defense genes following soil-soak inoculation with *Ralstonia solanacearum* strains GMI1000 or UW551 in susceptible cultivar Bonny Best or horizontally resistant line H7996. Genes represent activation of the jasmonic acid (JA) pathway (**A**: *Pin2*, **B**: *LoxA*), the ethylene (ET) pathway (**C**: *PR-1b*, **D**: *Osm*), and the salicylic acid (SA) pathway (**E**: *GluA*, **F**: *PR-1a*). Gene expression was measured by qRT-PCR in response to three pathogen cell densities: 1×10^7^ CFU/g stem (symptomless plants, white bars), 3×10^8^ CFU/g (symptomless or first wilting signs, grey bars), and 1×10^9^ CFU/g (early disease corresponding to DI = 1, black bars). Asterisks above bars indicate significant differences (*P*>0.05) in gene expression between mock and *R. solanacearum* inoculated tomatoes. *P*-values reflecting differences between cell densities (CFU), tomato cultivars and strains are shown in Table S2. Bars show normalized mean fold induction relative to mock-inoculated control plants (± SE). N = 6 to 12 plants for each cell density and strain, >3 independent experiments.

Quantitative RT-PCR gene expression analysis in susceptible and resistant tomato plants infected with *R. solanacearum* revealed little or no activation of the jasmonic acid (JA) pathway marker genes *Pin-2* and *LoxA* [18], [19]. However, both *PR-1b* and *Osm*, which are ET-induced [20], [21], [22], and *GluA* and *PR-1a*, which are regulated by the SA pathway [20], [22], [23], were expressed at significantly higher levels in plants with pathogen cell densities ≥3×10^8^ CFU/g, relative to water-inoculated controls (Figure 2).

### Resistant tomato plants activated the SA and ET defense pathways more rapidly than a susceptible cultivar

BW-resistant H7996 responded to large populations of both *R. solanacearum* strains by increasing expression of genes in the ET and SA signaling pathways by two to three orders of magnitude (Figure 2). Defense genes in H7996 were noticeably induced even at lower pathogen cell densities (1×10^7^ CFU of GMI1000/gm stem and 3×10^8^ CFU of UW551/gm stem). In contrast, susceptible cv. Bonny Best had no detectable defense response to 1×10^7^ CFU/gm. This result is consistent with the general observation that disease-resistant plants have faster and stronger defense responses [24].

### Large populations of strain GMI1000 triggered strong defense pathway gene expression in both susceptible and resistant tomato plants

*R. solanacearum* GMI1000, a broad host range tropical strain originally isolated from tomato, readily infected susceptible cv. Bonny Best. Resistant H7996 was less frequently infected and disease developed more slowly in this line, as is characteristic of horizontal resistance. However, when either Bonny Best or H7996 plants contained 1×10^9^ CFU of GMI1000/g stem, populations typical of full-blown wilt disease, their expression of *PR-1b* and *Osm* (ET pathway) and *GluA* and *PR-1a* (SA pathway) was two to four orders of magnitude larger than in plants at an early stage of colonization, containing just 1×10^7^ CFU/gm (Figure 2). This result suggests that GMI1000 induces similar defense responses in both susceptible and resistant tomato, but that the timing of response is different in the two hosts.

### Strain UW551 was able to avoid or calm defense responses in a susceptible tomato cultivar, but did activate defense gene transcription in BW resistant line H7996

We observed a strikingly different pattern of tomato responses to strain UW551, a temperate strain with a relatively narrow host range limited to potato, tomato, and some related species. At high bacterial cell densities in resistant H7996, UW551 elicited *PR-1a, Osm* and *GluA* expression levels similar to those induced by GMI1000. Only *PR-1b* expression was two orders of magnitude lower after infection with UW551 compared to GMI1000 (Figure 2). However, in susceptible Bonny Best UW551 had remarkably little effect on defense gene expression, which was two to three orders of magnitude lower than that elicited by GMI1000. Induction of *GluA*, representative of SA pathway activation, was especially weak (Figure 2E).

### Bacterial EPS plays different roles in susceptible and resistant tomato host responses

**Figure 3:**
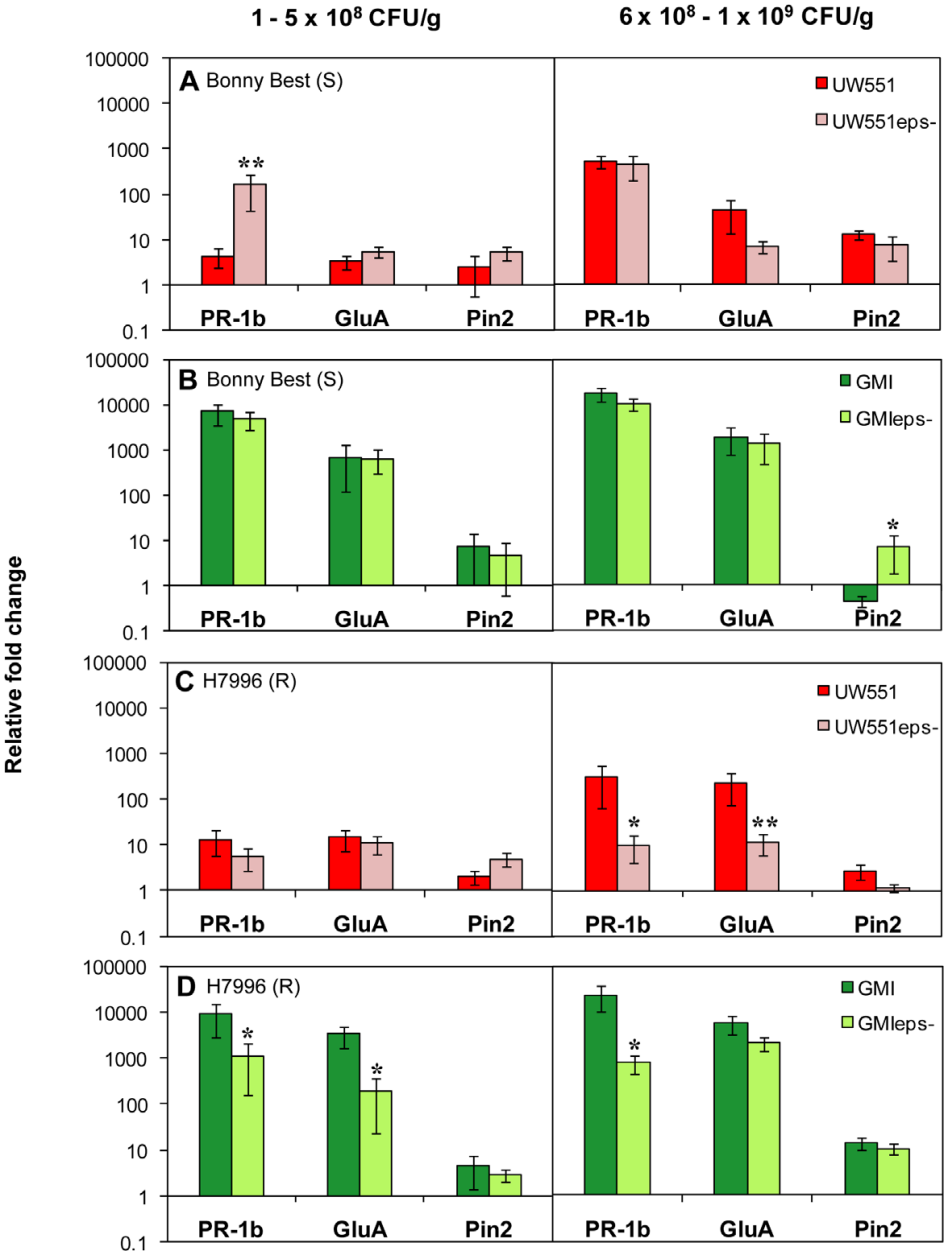
Expression of tomato defense genes following petiole inoculation with *Ralstonia solanacearum* wild-type strains and extracellular polysaccharide-deficient Δ*epsB* mutants. Gene expression was measured in **A**: BW-susceptible (S) cv. Bonny Best infected with UW551 or UW551Δ*epsB*; **B**: BW-susceptible cv. Bonny Best infected with GMI1000 or GMI1000Δ*epsB*; and **C**: horizontally resistant (R) line H7996 infected with UW551 or UW551Δ*epsB*; **D**: horizontally resistant line H7996 infected with GMI1000 or GMI1000Δ*epsB*. Plants were inoculated through the cut petiole of the first true leaf. Genes represent activation of ET pathway (*PR-1b*), SA pathway (*GluA*), and JA pathway (*Pin2*). Gene expression was measured in response to two pathogen cell densities in tomato stem tissue: 1 to 5×10^8^ CFU/g stem and 6×10^8^ to1×10^9^ CFU/g. Asterisks above bars indicate significant differences in gene expression between wild-type strain and Δ*epsB* mutant (*  =  *P*>0.05, **  =  *P* = 0.001). Bars show normalized mean fold induction relative to mock-inoculated control plants (± SE). UW551: N = 8 to 15 plants per treatment, with 4 independent experiments; GMI1000: N = 6 to 11 plants per treatment, with 3 independent experiments.

As documented for other *R. solanacearum* strains, an EPS-deficient mutant of UW551, UW551Δ*epsB*, was dramatically reduced in virulence on both susceptible and resistant tomato plants (*P*<0.001) and rarely killed the host (Figure S1). To test the hypothesis that EPS cloaks *R. solanacearum* from recognition by its plant host, we measured tomato defense gene expression following infection by wild-type and Δ*epsB* strains of the pathogen. The defense-associated *PR-1b* gene of susceptible Bonny Best was upregulated 40-fold (*P* = 0.001) in response to an *eps* ^−^ mutant of *R. solanacearum* UW551 compared to *PR-1b* expression triggered by wild-type bacteria. This finding is consistent with the cloaking hypothesis. This effect was observed up to a pathogen cell density of about 5×10^8^ CFU/g stem (Figure 3A, left). However, at higher cell densities (>5×10^8^ CFU/gm stem) led to increased defense gene expression (Fig. 3A, right). However, infection with wild-type and EPS-deficient strain GMI1000 elicited defense gene expression at comparable magnitudes in susceptible Bonny Best (Figure 3B).

Surprisingly, the opposite was true in BW resistant H7996 tomato plants. At cell densities below 5×10^8^ CFU/gm stem, the host responded to infections with wild-type and *eps^−^ R. solanacearum* strain UW551 by slightly upregulating tomato defense genes (Figure 3C, left). But when the pathogen exceeded 5×10^8^ CFU/gm stem, the wild-type strain elicited 30-fold higher *PR-1b* (*P* = 0.03) and 20-fold higher *GluA* expression (*P* = 0.001) than did the Δ*epsB* mutant (Figure 3C, right). Similarly, wild-type strain GMI1000 triggered a significantly stronger response than the Δ*epsB* mutant at all pathogen concentration tested. Even at 1×10^5^ CFU/g stem *PR-1b* expression was 8-fold higher (*P* = 0.049) and *GluA* expression showed an 18-fold increase (*P* = 0.015) after infection with the wild-type strain compared to the EPS-deficient strain. At cell densities above 5×10^8^ CFU/gm stem, the effect of EPS on defense gene expression became even more apparent since the wild-type strain elicited 30-fold higher *PR-1b* expression (*P* = 0.04) than GMI1000Δ*epsB*. Collectively, these results suggested that the resistant tomato can recognize the EPS produced by *R. solanacearum*.

### Resistant plants recognized cell-free EPS

**Figure 4:**
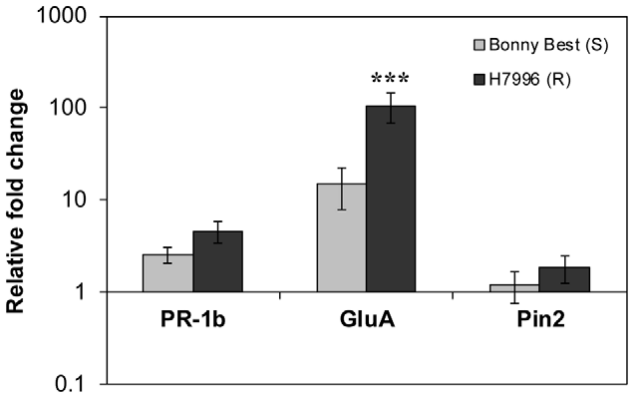
Expression of tomato defense genes in response to purified *Ralstonia solanacearum* extracellular polysaccharide. Gene expression was measured in bacterial wilt-susceptible cv. Bonny Best (S) and horizontally resistant line H7996 (R) by qRT-PCR 24 h after injection of 20 µg purified EPS through the cut petiole of the first true leaf directly into the vascular system. Genes represent activation of the ET pathway (*PR-1b*), the SA pathway (*GluA*), and the JA pathway (*Pin2*). Asterisks above bars indicate significant differences in gene expression between BW susceptible Bonny Best and horizontally resistant H7996 (***  =  *P*>0.0001). Bars show normalized mean fold induction relative to mock-inoculated control plants (± SE). N = 40 plants per treatment, in four independent experiments.

Plant defense expression levels could be affected by the effectors and enzymes secreted by live bacteria. To more directly test the hypothesis that pathogen EPS triggers defense responses in wilt-resistant plants, we measured tomato transcriptional response to a biologically relevant amount (20 µg) [9] of extensively purified EPS from UW551. Purified EPS activated the SA pathway (*GluA*) in H7996 to a significantly greater degree (7-fold, *P* = 0.00003) than in Bonny Best. This indicates that the resistant host perceives and responds to *R. solancearum* EPS. Interestingly, although live cells of the wild-type pathogen triggered much higher *PR-1b* expression in H7996 than UW551Δ*epsB* did, cell-free EPS alone did not significantly increase *PR-1b* expression, suggesting that EPS activates only a subset of defense-associated responses (Figure 4). Alternatively, full-spectrum signal transduction may require interaction of EPS with specific tissues in ways that occur during natural infection but not when EPS is introduced directly into the stem.

### EPS triggered a strong oxidative burst in resistant plants

**Figure 5:**
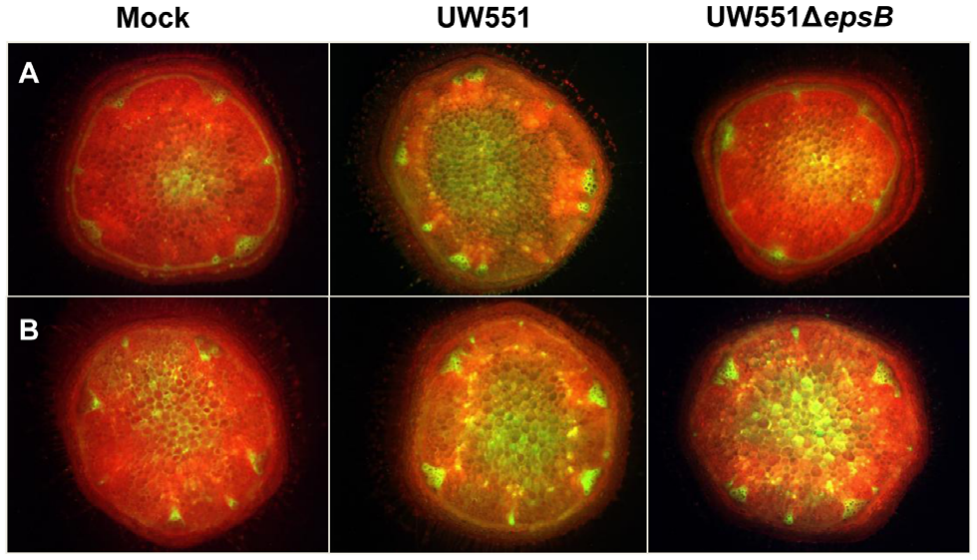
Accumulation of reactive oxygen species (ROS) in tomato stem tissue. ROS were determined in **A**: horizontally BW-resistant tomato H7996 and **B**: susceptible cv. Bonny Best 48 h after infection with *Ralstonia solanacearum* wild-type strain UW551 or UW551Δ*epsB* or water (mock-inoculated control). At 48 h post-inoculation, stem cross-sections containing 10^5^ CFU/g bacteria were stained with 50 µM dihydrorhodamine 123 (DHR 123) and fluorescence microscopy was used to visualize the green fluorescence of rhodamine 123 generated by oxidizing DHR 123 by ROS. Three independent experiments each contained eight plants per treatment; representative results are shown.

**Figure 6:**
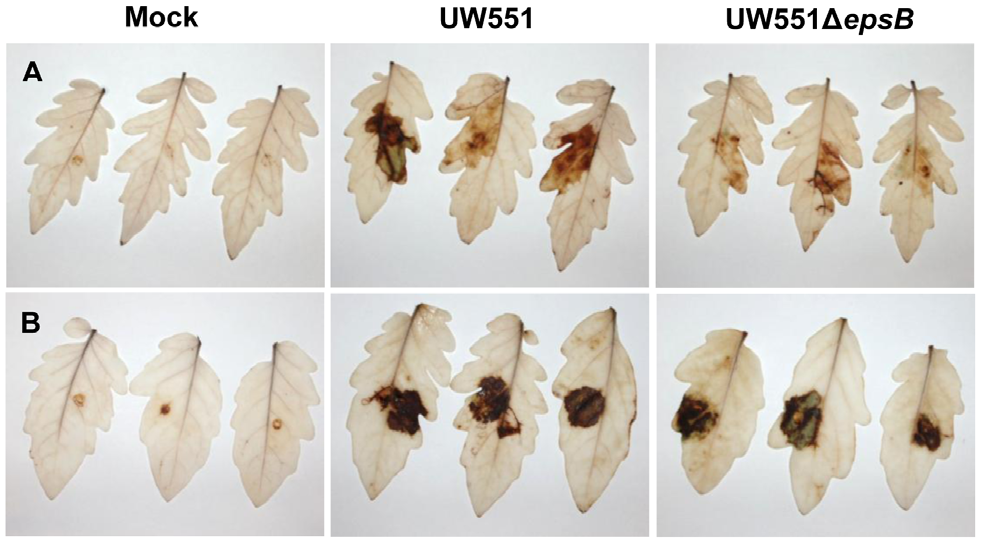
Reactive oxygen species (ROS) accumulation in tomato leaves. ROS were determined in **A**: horizontally BW-resistant line H7996 and **B**: BW-susceptible cv. Bonny Best 48 h after infusion with 1×10^9^ CFU/ml *R. solanacearum* strain UW551 or EPS I mutant UW551Δ*epsB*. ROS appears as a brown precipitate after leaves were stained with the *in situ* endogenous peroxidase-dependent histochemical stain 3,3′-diaminobenzidine (DAB). The experiment was repeated five times; representative results are shown.

To determine if the defense-associated gene expression patterns we observed in response to wild-type and EPS-deficient *R. solanacearum* cells correlated with biochemical indicators of active plant defenses, we used the fluorescent dye dihydrorhodamine123 to assess tomato stem levels of ROS, a common element of plant antimicrobial defenses [1]. This qualitative dye revealed that infection by wild-type *R. solanacearum* UW551 triggered a strong oxidative burst in the vascular bundles of both resistant and susceptible tomato plants (Figure 5). In contrast, H7996 plants infected with 10^4^ to 10^5^ CFU/g of UW551Δ*epsB* accumulated noticeably less ROS than did H7996 stems carrying similar populations of wild-type *R. solanacearum* (Figure 5A). No such response was observed in cv. Bonny Best, where stems containing 10^4^ to 10^5^ CFU/g of UW551Δ*epsB* had ROS levels indistinguishable from those in stems infected by the wild-type strain (Figure 5B). These differences in ROS accumulation triggered by wild-type and EPS-deficient bacteria were also seen in tomato leaves, indicating that this phenomenon is not unique to stem tissue (Figure 6).

## Discussion

Host resistance is the optimal strategy for controlling BW disease, but the specific triggers and mechanisms responsible for horizontal wilt resistance in tomato are not known. We found that the tomato ET and SA signaling pathways are activated during BW disease resistance. This is consistent with the finding that VIGS-mediated disruption of the JA, ET, SA and MAPK pathways increased colonization of stem bases and/or mid-stems in H7996 by *R. solanacearum* strain Pss4 [25], [26]. In addition, overexpressing the ET pathway decreased wilting symptoms in susceptible L390 tomato [25]. These results suggested that the JA, ET and SA defense signaling pathways interact synergistically in the resistance of tomato against BW. However, under our experimental conditions, JA pathway marker genes were not substantially upregulated in response to either *R. solanacearum* strain in resistant or in susceptible tomato plants.

Arabidopsis has been used as model plant for the study of *R. solanacearum*-host interactions, but it is not a natural host of *R. solanacearum* and artifactual inoculation methods are required to generate symptoms [27]. Our results and those of others [28] suggest that this model plant may react differently to *R. solanacearum* than the natural host tomato. Disease development and proliferation of GMI1000 in Arabidopsis was not SA-dependent, but inactivation of ET-related signaling pathways resulted in decreased symptom development in susceptible plants, indicating that ET-regulated defenses reduce disease severity [29]. In contrast, resistance of Arabidopsis ecotype Nd-1, which unlike tomato carries a single vertical resistance gene (*RSS1*), was partially dependent on SA [30], but appeared to be independent of ET signaling [29]. Further, the JA signaling pathway may suppress Arabidopsis defense against *R. solanacearum*, since JA-insensitive Arabidopsis plants displayed milder disease symptoms [31]. Overall, the responses of Arabidopsis plants to *R. solanacearum* appear to differ significantly from those of tomato.

We describe here the kinetics of tomato defense gene expression against two biologically distinct *R. solanacearum* strains in BW-resistant and susceptible hosts. Our results are consistent with the general observation that major differences between resistant and susceptible responses are quantitative and/or kinetic, and not necessarily caused by the expression of different sets of genes [32], [33]. H7996 resistance was characterized by a faster response kinetic; the ET and SA pathways were activated at much lower threshold pathogen cell densities in H9776 xylem than in susceptible Bonny Best. The ultimate magnitude of the plant response to high pathogen cell densities was comparable between susceptible and resistant plants, but it differed strikingly between the two strains. In particular, susceptible Bonny Best launched stronger defenses against strain GMI1000 than against strain UW551.

Genetic differences between the strains could explain why these two pathogen strains trigger different responses from the plant host. The GMI1000 genome contains about 1000 coding sequences not present in UW551, while about 500 genes are unique to UW551 [34]; most of these genes encode hypothetical proteins. The products of any of those strain-specific genes, or differential regulation of common genes, could lead to differential recognition of the pathogen and might explain how temperate strain UW551 calms or evades host recognition. One likely explanation is that strain UW551 deploys Type 3-secreted (Hrp) effectors that specifically suppress defense responses in Bonny Best. *R. solanacearum* strains do produce Type 3-secreted effectors that reduce plant innate immunity [14], and indeed we found that expression of genes in the ET and SA signaling pathways was reduced in tomato plants infected by a *hrp* mutant (A. Milling and J. M. Jacobs, unpublished results). Further, a recent *in planta* microarray analysis in our lab revealed expression trend differences between UW551 and GMI1000. Of 31 orthologous genes encoding Type 3-secreted effectors or HrpB-dependent secretion system structural components, 25 were upregulated to a significantly greater degree in UW551 than in GMI1000 (Jacobs et al., in preparation). This is an intriguing topic for future study.

Interestingly, although strain GMI1000 triggered stronger expression of the ET and SA pathway genes than UW551, these strains induced indistinguishable rapid disease progress in susceptible tomato plants. In contrast, the resistance of H7996 to GMI1000 may result from more rapid induction of the ET and SA pathways. It seems likely that the differential expression of defense signaling pathways we observed in Bonny Best and H7996 is accompanied by expression of diverse additional plant genes that confer specific aspects of wilt susceptibility or tolerance.

*R. solanacearum's* nitrogen- and carbohydrate-rich EPS is metabolically expensive and its production is tightly regulated by a complex network. Nonetheless, it is abundantly produced at high cell densities and inside host plants [9], [13] and it is critical for bacterial wilt virulence [16]. Why? It has been hypothesized that EPS protects *R. solanacearum* from plant antimicrobial defenses by cloaking bacterial surface features from host recognition. It would seem advantageous for hosts to recognize an abundantly expressed extracellular molecule required for virulence. However, bacterial EPS is generally not perceived by eukaryotes as a MAMP, but rather enables bacteria to evade immunity [35]. We did observe that susceptible plants upregulated the ET pathway to a greater degree in response to an *eps^−^* mutant of one *R. solanacearum* strain, temperate R3bv2 strain UW551, at least at lower pathogen cell densities. Overall, however, the susceptible cultivar responded similarly to wild-type and *eps^−^* strains, which does not support the cloaking hypothesis.

It has been suggested that the EPS of many bacterial plant pathogens, including *R. solanacearum*, non-specifically suppresses MAMP-triggered immunity via sequestration of apoplastic calcium ions, which play a role in defense signaling [36]. However, we found that *R. solanacearum* EPS does not suppress plant defenses, but rather plays a more specific role in inducing plant defenses. Our experiments with *eps^−^* mutants and with purified EPS from two different *R. solanacearum* strains demonstrate that EPS can specifically elicit defense gene expression and ROS production in at least one resistant tomato genotype. The SA defense signaling pathway in H7996 appears especially responsive to EPS-induced signaling. The susceptible cultivar generally responded similarly to wild-type and EPS-deficient strains, which is also inconsistent with the calcium hypothesis.

EPS is a virulence factor for many plant pathogenic bacteria, and the chemical structures of these polysaccharides vary among species, suggesting diversifying selection pressure [15]. Some experiments have suggested that certain plants can recognize EPS from specific bacteria. Potato cultivars have membrane-bound receptors that recognize EPS from *Clavibacter michiganensis* pv. *sepedonicus* and induce defense responses [37]. EPS extracts from *Pseudomonas syringae* pv. *ciccaronei* and *P. savastonoi* pv. *nerii* caused necrotic lesions when infiltrated into tobacco leaves, induced H_2_O_2_ release from tobacco cells in culture medium, and decreased cytosolic ascorbate peroxidase (APX), one of the main enzymes for ROS scavenging in plant cells; EPS extracts from the related plant pathogen *P. caryophylli* had no such effects [38]. EPS from an incompatible isolate of *Xanthomonas campestris* pv. *vesicatoria* elicited phytoalexin production in pepper leaves [39]. Moreover, the EPS produced by bacteria present in the mammalian gut can increase certain host immune responses [40], [41].

Recognition of *R. solanacearum* EPS, either specifically or as a MAMP, could give BW-resistant H7996 tomato plants a crucial advantage by triggering faster defense responses. To evade host recognition, plant pathogenic bacteria are known to vary MAMP structure both within and across species [27], [42]. Our result suggests that polymorphisms also exist on the host side for elicitor perception. Bacterial wilt resistance in H7996 is polygenic and complex, so EPS-triggered defenses can explain only part of its resistance. Nonetheless, if H7996 proves unique among tomato lines in its ability to perceive *R. solanacearum* EPS, this may explain why it has consistently ranked as the most wilt resistant tomato line in multiple comparative field trials [5], [43], [44].

Identifying the presumptive EPS receptor could elucidate the mechanism by which H7996 recognizes EPS. However, it is unclear which and how many additional *R. solanacearum* elicitors or MAMPs are detected by the tomato host. Insights acquired through expression profiling of single genes as presented here are necessarily incomplete. A more comprehensive microarray analysis could monitor global transcriptional responses to *R. solanacearum* in susceptible and resistant tomato hosts to generate a broader understanding of BW resistance and identify targets for marker-assisted breeding of wilt-resistant plants.

## Materials and Methods

### Bacterial strains and growth conditions

*R. solanacearum* strains used in these experiments were tropical strain GMI1000 (phylotype I, sequvar 18, biovar 3) [45], [46] and temperate strain UW551 (phylotype II, sequevar 1, biovar 2, historically known as Race 3) [17], [34]. To facilitate enumeration of *R. solanacearum* in the natural plant microbial background, all inoculations were performed with rifampicin-resistant *R. solanacearum* strains [47]. We confirmed that the rif-resistant strains had wild-type virulence and elicited plant gene expression comparable to the wild-type strain. *R. solanacearum* was grown on CPG solid medium [48] at 28°C for 48 h. If required, antibiotics were added at final concentrations of 25 mg/l kanamycin and 25 mg/l rifampicin. Medium components were from Difco Laboratories (Detroit, MI). All other chemicals and antibiotics were from Sigma-Aldrich (St. Louis, MO) or Fisher Scientific (Hanover Park, IL).

### Construction of an *eps^−^* mutant

An approximately 2,000-bp internal DNA fragment of the *epsB* gene of *R. solanacearum* strain UW551 (RRSL_1061) was amplified by PCR using primers UW551EPSBi-F: 5′-GACGAATTCGTCAGCTTCTTGGGCTTCAC and UW551EPSBi-R: 5′-GACTCTAGACTCAAGACGCTGGAGATGCT. To delete *epsB*, this amplicon was digested with *Eco*R1 and *Xba*I and ligated into the suicide vector pVIK112 [49] to create pEPSBi. This construct was moved into UW551 by conjugation with selection for kanamycin resistance. Correct deletion of *epsB* was verified by PCR and by comparing colony morphologies of wild-type and mutant strains. Extraction of cell-free EPS confirmed that UW551Δ*epsB* produced less than 5% of the EPS made by wild-type UW551. The corresponding mutant was constructed in strain GMI1000 by moving the Δ*epsB* construct into the GMI1000 genome by natural transformation. Correct deletion of the gene was verified by PCR and the *eps^−^* phenotype was verified by colony morphology.

### Plant inoculations and tissue collection

We compared the virulence of *R. solanacearum* strains GMI1000 and UW551 in susceptible cultivar Bonny Best and horizontally resistant line Hawaii7996 (H7996) by means of a naturalistic soil soak inoculation [50]. Briefly, unwounded 19 to 21 day old plants were inoculated by pouring a bacterial suspension onto the soil to a final density of approximately 1×10^8^ CFU/g soil, followed by incubation at 28°C. Control plants were mock-inoculated with sterile water. Symptoms were scored daily by a rater blind to treatment identity on a 0-to-4 disease index, where 0 indicates no disease, 1 indicates 1 to 25% of leaves wilted, 2 indicates 25 to 50% of leaves wilted, 3 indicates 51 to 75% of leaves wilted, and 4 indicates 76 to 100% of leaves wilted. Each experiment contained 16 plants per treatment, and experiments were repeated at least three times. To measure plant gene expression, Bonny Best tissue was sampled 4 to 7 dpi, while H7996 samples were collected 7 to 14 dpi due to slower disease development in this resistant host.

We measured disease progress and host defense responses to wild-type strains UW551 and GMI1000 and to UW551Δ*epsB* and GMI1000Δ*epsB* in the two tomato cultivars by directly inoculating 21-day old plants with either *R. solanacearum* wild-type (2×10^3^ cells) or Δ*epsB* (2×10^5^ cells) through the cut petiole of the first true leaf. Higher inoculum levels were necessary for the Δ*epsB* strains because of their reduced colonization rate. Control plants were inoculated with sterile water. Samples were taken 3 to 4 dpi from plant stems containing 1×10^8^ to 1×10^9^ CFU/g *R. solanacearum*.

### RNA extraction

Samples (100 mg) from randomly selected individual tomato plants were taken from mid-stem just above the cotyledon. One sub-sample was ground in sterile water, and dilution plated in triplicate to determine pathogen population size in the plant. Colonies were counted after 48 h incubation at 28°C. Another sub-sample was immersed in RNA*later* (Ambion Inc., Austin, TX) for 24 h at 4°C to preserve RNA integrity before storage at −80°C. Ultimately, RNA was extracted from tomato samples that contained the target bacterial cell densities of about 1×10^7^ CFU/g (symptomless plants) and 1×10^8^ or 1×10^9^ CFU/g (disease index 1, early disease) using the RNeasy Plant Mini Kit (Qiagen, Valencia, CA) including DNaseI treatment according to the manufacturer's instructions. RNA quantity and quality were assessed with micro-spectrophotometry (NanoDrop Technologies Inc., Wilmington, DE).

### Gene expression analysis using quantitative real-time PCR

To measure plant mRNA levels, 1 µg of total RNA from each sample was reverse transcribed into cDNA using Superscript III reverse transcriptase First-Strand Synthesis SuperMix (Invitrogen, Carlsbad, CA) containing oligo (dT) and random hexamer primers according to the manufacturer's instructions. Quantitative RT-PCR primers for selected tomato defense genes and three constitutively expressed normalization genes (Table S1) were designed using Biology Workbench software from the relevant GenBank (NCBI) tomato mRNA sequences. Quantitative RT-PCR amplifications were performed in duplicate 25 µl reactions using PowerSYBR Green Mastermix (Applied Biosystems, Warrington, UK) and consisted of 1X Mastermix, 400 nM forward and reverse primer, and 50 ng template cDNA. Reactions were run on an ABI PRISM 7300 Real-Time PCR System (Applied Biosystems, Foster, CA). Reaction parameters were: 10 min polymerase activation, followed by 40 cycles of 95°C for 15 s and 57°C for 1 min. Gene expression was quantified separately for each cDNA sample. Controls were cDNA samples lacking reverse transcriptase to check for DNA contamination and no-template reactions. Reaction efficiencies were between 94 and 105% for all primers, calculated by generating a standard curve and plotting the threshold cycle (C_T_) against the logarithm of four known tomato DNA dilutions. The number of cycles at threshold level was converted to relative quantities (RQ) with the highest expression set to one using the delta-C_T_ formula RQ  =  E^(minC^_T_^ – sampleC^_T_^)^ [51]. For maximum accuracy and reliability, RQ was divided by a normalization factor derived from the geometric mean of three reference genes, *Gapdh, Actin*, and *DnaJ*-like protein, to generate normalized relative quantities (NRQ) [51], [52]. Stability of the reference transcripts was validated using geNorm, and normalization factors were calculated in the geNorm applet [51]. Relative expression change was calculated by calibrating treated (infected) samples to the mean NRQ of at least three control replicates within each experiment. Data are presented as fold change in defense gene expression in infected tomato plants relative to mock-inoculated control plants. Each experiment was replicated at least three times.

### Experiments with extracellular polysaccharide (EPS I)

EPS I was extracted from *R. solanacearum* strain UW551 and extensively purified using a modification of a described protocol [9]. Bacterial cells were scraped from the surface of CPG agar medium, resuspended in water to an O.D_600_ of 1.0 and centrifuged for 10 min twice at 8000 rpm. The cell-free supernatant was lyophilized and redissolved in 10 ml distilled water. EPS I was precipitated overnight at −20°C using 4 vol acetone and 20 mM NaCl and redissolved in DNaseI buffer (50 mM Tris, 1 mM MgCl_2_). DNaseI (Roche Diagnostics, Indianapolis, IN) was added to a final concentration of 0.1 g/ml and the solution was incubated at 37°C for 1 h and extracted once with phenol, followed by successive extractions with chloroform until no interphase was visible. The aqueous layer was dialyzed extensively against distilled water. Pure EPS was recovered from the dialysate by overnight precipitation with 3 vol ethanol at −20°C. The purified EPS was air-dried, dissolved in 250 µl distilled water and frozen in aliquots until use. EPS was quantified by the Elson-Morgan assay for hexosamine sugars using N-acetylgalactosamine as the standard [53], [54]. Protein content was estimated using the Pierce BCA assay kit (ThermoScientific, Rockford, IL) with BSA as the standard, and nucleic acid content was estimated micro-spectrophotometrically. Tomato gene expression in response to purified EPS was determined 24 h after injection of 20 µg EPS directly into the vascular system through the cut petiole. Control plants were injected with sterile water. Each EPS experiment contained 10 plants per treatment and experiments were repeated four times.

### ROS detection in tomato stem tissue

ROS accumulation in stems of susceptible cv. Bonny Best and horizontally resistant line H7996 was monitored 48 h to 72 h after infection of plants with *R. solanacearum* UW551 or UW551Δ*epsB* via cut petiole as described above. Water inoculated plants served as controls. Plants were cut horizontally through the mid-stem and left at room temperature to release the first wave of wounding-related oxidative burst. After 15 min, a fresh cross section (1 mm thick, 5 mm diameter) was removed from the stem and incubated in the dark with 5 µl of a 50 µM dihydrorhodamine 123 solution (DHR123, AnaSpec Inc., Fremont, CA) for 30 min to allow ROS in the tissue to oxidize non-fluorescent DHR 123 to the fluorescent rhodamine 123. Fluorescence was observed with a Leica MZ FLIII fluorescence stereomicroscope using 480/40 nm (excitation) and a 510 nm barrier filter. Another stem section was used to quantify *R. solancearum* populations as described above to ensure comparison of fluorescence between samples containing similar bacterial populations. Each experiment contained 6 to 8 plants per treatment, and the experiment was repeated three times.

### Reactive oxygen species (ROS) detection in tomato leaves

ROS accumulation in host tissue was detected by endogenous peroxidase-dependent *in situ* histochemical staining with 3,3′-diaminobenzidine (DAB, Sigma, USA) using a slightly modified protocol [55]. Oxidation of DAB by ROS creates a visible brown precipitate in the host tissue. Eight tomato leaves from 28-day old tomato plants of BW-susceptible cv. Bonny Best and horizontally resistant line Hawaii7996 were infused with 1×10^9^ CFU/ml *R. solanacearum* cells or water as control. Three leaves were cut at the petiole 48 h after inoculation and immediately immersed in DAB solution (1 mg/ml in water, pH 3.8). Leaves were incubated for 18 h in the dark at room temperature, and then bleached in boiling 96% ethanol for 10 min, cleared and stored in 70% ethanol until imaging. The fourth leaf was used to quantify *R. solancearum* populations in the leaf. Three disks (5 mm diameter) per leaf were pooled, ground in sterile water, serially diluted and plated on CPG solid medium in triplicate. Colonies were counted after 48 h incubation at 28°C. The experiment was repeated five times with comparable results.

### Data analysis

The log2 of NRQs of each plant defense gene tested was used to analyze differences in gene expression caused by infection with *R. solanacearum* compared to untreated water controls. The log2 of fold change was used to compare gene expression elicited by strains GMI1000 and UW551, as well as expression elicited by strains UW551 and UW551Δ*epsB* [56]. Data were analyzed by ANOVA using the GLM procedure. Specific comparisons of least-square means were evaluated for significance using Turkey's HSD adjusted *P*-values (Table S2). Gene expression levels elicited by purified EPS I were compared using a 2-tailed *t*-test. Repeated measures ANOVA using the PROC mixed method was used to compare disease progress curves of diverse strains in resistant and susceptible tomato plants. These analyses were conducted in SAS version 9.1 (SAS Institute, Cary, NC). A *P*-value of <0.05 was considered statistically significant.

## Supporting Information

Figure S1 **Virulence of wild-type** ***Ralstonia solanacearum*** **strain UW551 and EPS-deficient mutant UW551Δ** ***epsB*** **on resistant and susceptible tomato plants.** 21-day-old susceptible (cv. Bonny Best) and horizontally resistant (H7996) tomato plants were inoculated **A**: by pouring bacteria onto the soil to a final concentration of about 1×10^8^ CFU/g soil or **B**: with 2000 cells via the cut petiole of the first true leaf followed by incubation in a 28°C growth chamber. Plants were rated daily over 14 days on a disease index scale from 0 to 4 where 0 indicated healthy and 4 indicated 100% wilted. Each point represents the mean disease index for three independent experiments each with 16 plants per treatment.
(TIF)

Table S1 **Primers used in the real-time qRT-PCR analysis of defense-related tomato genes.**
(DOC)

Table S2 **ANOVA results for gene expression elicited by** ***R. solanacearum*** **strain GMI1000 or UW551 in BW-susceptible tomato cultivar Bonny Best and horizontally resistant line.**
(DOC)
