# Supplementary figures and images for: Ralstonia solanacearum Extracellular Polysaccharide Is a Specific Elicitor of Defense Responses in Wilt-Resistant Tomato Plants
Source: PLoS One. 2011 Jan 6;6(1):e15853. doi: 10.1371/journal.pone.0015853 (PMC3017055; doi:10.1371/journal.pone.0015853)

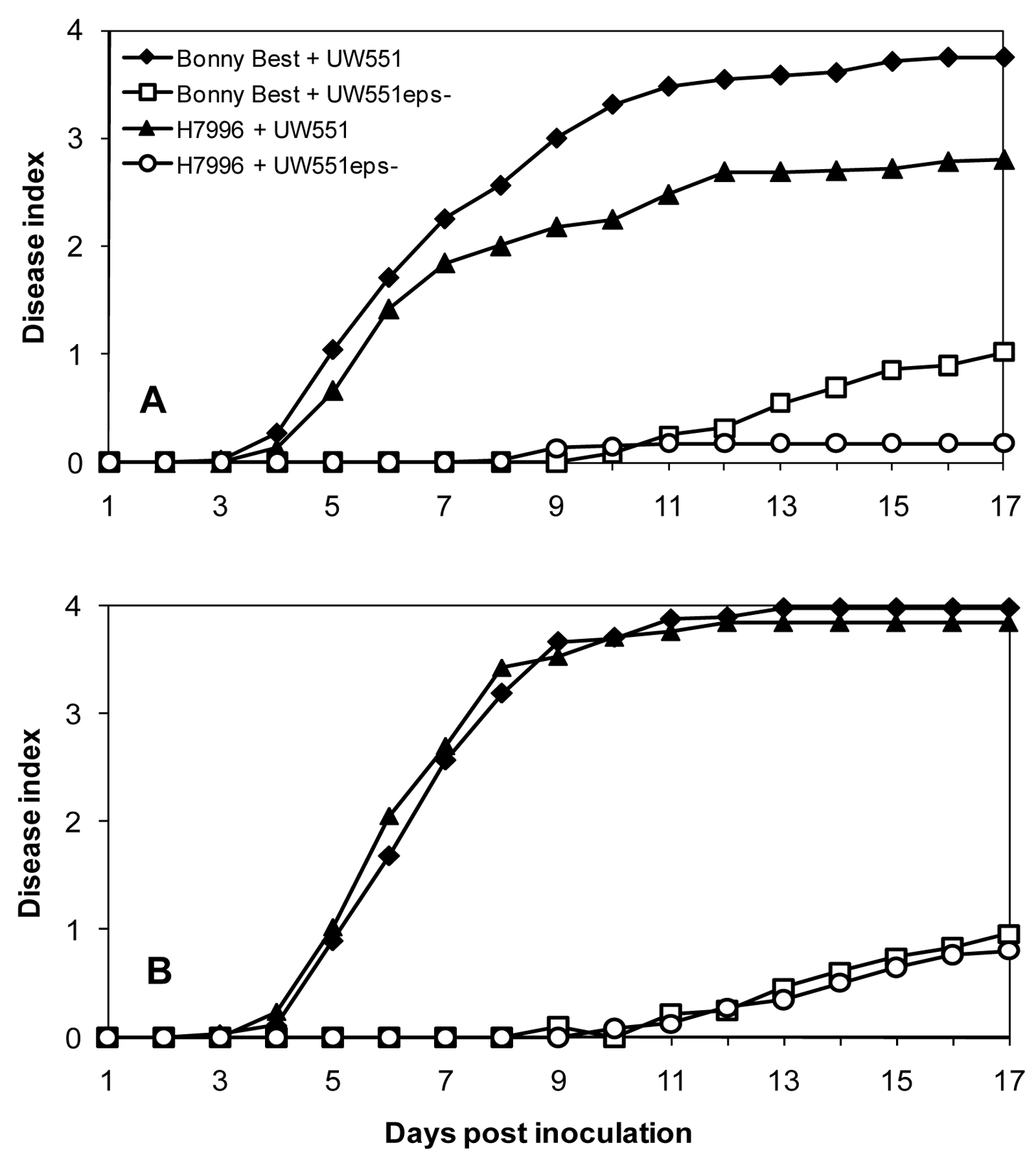

Supplement: Figure S1 — Virulence of wild-type Ralstonia solanacearum strain UW551 and EPS-deficient mutant UW551Δ epsB on resistant and susceptible tomato plants. 21-day-old susceptible (cv. Bonny Best) and horizontally resistant (H7996) tomato plants were inoculated A: by pouring bacteria onto the soil to a final concentration of about 1×10^8^ CFU/g soil or B: with 2000 cells via the cut petiole of the first true leaf followed by incubation in a 28°C growth chamber. Plants were rated daily over 14 days on a disease index scale from 0 to 4 where 0 indicated healthy and 4 indicated 100% wilted. Each point represents the mean disease index for three independent experiments each with 16 plants per treatment. (TIF) [file pone.0015853.s001.tif]
