# Supplementary material for: Ralstonia solanacearum Extracellular Polysaccharide Is a Specific Elicitor of Defense Responses in Wilt-Resistant Tomato Plants
Source: PLoS One. 2011 Jan 6;6(1):e15853. doi: 10.1371/journal.pone.0015853 (PMC3017055; doi:10.1371/journal.pone.0015853)
Supplement: Table S1 — Primers used in the real-time qRT-PCR analysis of defense-related tomato genes. (DOC) [file pone.0015853.s002.doc]

**Table S1.** Primers used in the real-time qRT-PCR analysis of defense related tomato genes

| Gene | Pathway^a^ | GenBank  accession | Sequence (5’-3’)^b^ | Reference |
| --- | --- | --- | --- | --- |
| *Pin2* | JA | AY129402 | F - TGATGCCAAGGCTTGTACTAGAGA  R - AGCGGACTTCCTTCTGAACGT | this study |
| *LoxA* | JA | U09026 | F - TGGTAGACCACCAACACGAA  R - GACCAAAACGCTCGTCTCTC | this study |
| *PR-1b* | ET | X14065 | F - TTGGTGACTGCGGGATGA  R - GGCGGCGGCTAGGTT T | this study |
| *Osmotin*-like | ET | M21346 | F - TGTACCACGTTTGGAGGACA  R - ACCAGGGCAAGTAAATGTGC | this study |
| *GluA* | SA | M80604 | F - TCA GCA GGG TTG CAA AAT CA  R - CTCTAGGTGGGTAGGTGTTGGTTAA | this study |
| *PR-1a* | SA | M69247 | F - GAGGGCAGCCGTGCAA  R - CACATTTTTCCACCAACACATTG | [22] |
| *Actin* | HK | BT013524 | F - TCAGCAACTGGGATGATATG  R - TTAGGGTTGAGAGGTGCTTC | this study |
| *Gapdh* | HK | U93208 | F - CTCCATCACAGCCACTCAGA  R - TTCCACCTCTCCAATCCTTG | this study |
| *DnaJ*-like | HK | AF124139 | F - ATGAAGCGCCAGATACCATC  R - TCAAGGCTCAATGTGTGCTC | this study |

^a^ Gene representative for signaling pathway: JA- jasmonic acid, ET – ethylene, SA – salicylic acid, HK – housekeeping gene for normalization

^b^ Primers were designed using the primer3 program from Biology workbench and its specificity for the corresponding gene was confirmed by blast search at www.ncbi.nlm.nih.org database.
