# Supplementary material for: Ralstonia solanacearum Extracellular Polysaccharide Is a Specific Elicitor of Defense Responses in Wilt-Resistant Tomato Plants
Source: PLoS One. 2011 Jan 6;6(1):e15853. doi: 10.1371/journal.pone.0015853 (PMC3017055; doi:10.1371/journal.pone.0015853)
Supplement: Table S2 — ANOVA results for gene expression elicited by R. solanacearum strain GMI1000 or UW551 in BW-susceptible tomato cultivar Bonny Best and horizontally resistant line. (DOC) [file pone.0015853.s003.doc]

**Table S2.** ANOVA results for gene expression elicited by *R. solanacearum* strain GMI1000 or UW551 in BW-susceptible tomato cultivar Bonny Best and horizontally resistant line H7996

| Gene | Effect | df^a^ | *F* value | *P* value^b^ |
| --- | --- | --- | --- | --- |
| *Pin2* | CFU | 2 | 9.34 | 0.0003 |
|  | Tomato | 1 | 7.25 | 0.0095 |
|  | Strain | 1 | 43.87 | < 0.0001 |
|  | CFU x Tomato | 2 | 2.27 | 0.1136 ns |
|  | CFU x Strain | 2 | 0.43 | 0.6506 ns |
|  | Tomato x Strain | 1 | 2.72 | 0.1053 ns |
|  |  |  |  |  |
| *LoxA* | CFU | 2 | 15.65 | < 0.0001 |
|  | Tomato | 1 | 83.98 | < 0.0001 |
|  | Strain | 1 | 15.66 | 0.0001 |
|  | CFU x Tomato | 2 | 9.14 | 0.0002 |
|  | CFU x Strain | 2 | 2.37 | 0.0986 ns |
|  | Tomato x Strain | 1 | 4.51 | 0.0363 |
|  |  |  |  |  |
| *PR-1b* | CFU | 2 | 15.12 | < 0.0001 |
|  | Tomato | 1 | 5.13 | 0.0257 |
|  | Strain | 1 | 53.56 | < 0.0001 |
|  | CFU x Tomato | 2 | 1.80 | 0.1709 ns |
|  | CFU x Strain | 2 | 1.86 | 0.1616 ns |
|  | Tomato x Strain | 1 | 1.08 | 0.3007 ns |
|  |  |  |  |  |
| *Osm* | CFU | 2 | 9.34 | 0.0003 |
|  | Tomato | 1 | 7.25 | 0.0095 |
|  | Strain | 1 | 43.87 | < 0.0001 |
|  | CFU x Tomato | 2 | 2.27 | 0.1136 ns |
|  | CFU x Strain | 2 | 0.43 | 0.6506 ns |
|  | Tomato x Strain | 1 | 2.72 | 0.1053 ns |
|  |  |  |  |  |
| *GluA* | CFU | 2 | 11.82 | < 0.0001 |
|  | Tomato | 1 | 70.90 | < 0.0001 |
|  | Strain | 1 | 42.55 | < 0.0001 |
|  | CFU x Tomato | 2 | 0.49 | 0.6136 ns |
|  | CFU x Strain | 2 | 2.98 | 0.0557 |
|  | Tomato x Strain | 1 | 4.65 | 0.0336 |
|  |  |  |  |  |
| *PR-1a* | CFU | 2 | 17.20 | < 0.0001 |
|  | Tomato | 1 | 14.39 | 0.0003 |
|  | Strain | 1 | 37.07 | < 0.0001 |
|  | CFU x Tomato | 2 | 2.74 | 0.0707 ns |
|  | CFU x Strain | 2 | 0.86 | 0.4254 ns |
|  | Tomato x Strain | 1 | 5.25 | 0.0245 |

^a^ df, degrees of freedom

^b^ ns = not significant (*P* > 0.05)
